# Exosomes derived from human umbilical cord mesenchymal stem cells inhibit vein graft intimal hyperplasia and accelerate reendothelialization by enhancing endothelial function

**DOI:** 10.1186/s13287-020-01639-1

**Published:** 2020-03-23

**Authors:** Qingxi Qu, Yingxin Pang, Chunmei Zhang, Linghong Liu, Yanwen Bi

**Affiliations:** 1 Department of Obstetrics and Gynecology, Qilu Hospital of Shandong University, Jinan, 250012 Shandong People’s Republic of China; 2 Department of Cardiology, Qilu Hospital of Shandong University, Jinan, 250012 Shandong People’s Republic of China; 3 Research Center of Stem Cell and Regenerative Medicine, Shandong University, Jinan, 250012 Shandong People’s Republic of China; 4 Laboratory of Cryomedicine, Qilu Hospital of Shandong University, Jinan, 250012 Shandong People’s Republic of China; 5 Department of Cardiovascular Surgery, Qilu Hospital of Shandong University, Jinan, 250012 Shandong People’s Republic of China

**Keywords:** Exosomes, Mesenchymal stem cell, Vein graft, Reendothelialization, Neointimal hyperplasia

## Abstract

**Background:**

In our previous research, we found that mesenchymal stem cell (MSC) transplantation therapy can inhibit intimal hyperplasia and enhance endothelial function in arterialized vein grafts in rats. However, whether MSC-derived exosomes (MSC-exosomes) can reduce neointimal formation and its possible mechanism is still unclear.

**Methods:**

The primary human umbilical cord MSCs (hucMSCs) and human umbilical vein endothelial cells (HUVECs) were isolated and characterized by flow cytometry and immunofluorescence. The exosomes derived from hucMSCs (hucMSC-exosomes) were identified by transmission electron microscopy and western blots. hucMSC-exosomes were intravenously injected into a rat model of vein grafting, and its effect on vein grafts reendothelialization and intimal hyperplasia was assessed by physical, histological, immunohistochemistry, and immunofluorescence examinations. The effects of hucMSC-exosomes on endothelial cells were evaluated by integrated experiment, EdU staining, scratch assay, and Transwell assay. The expression levels of key gene and pathways associated with the biological activity of vascular endothelial cells were evaluated following the stimulation of hucMSC-exosomes.

**Results:**

We successfully isolated and characterized primary hucMSCs and hucMSC-exosomes and primary HUVECs. We verified that the systemic administration of hucMSC-exosomes accelerates reendothelialization and decreases intimal hyperplasia of autologous vein graft in a rat model. We also identified that hucMSC-exosomes can be uptaken by endothelial cells to stimulate cell proliferative and migratory activity in vitro. Furthermore, we detected that vascular endothelial growth factor (VEGF) plays an important part in hucMSC-exosome-mediated proliferation and migration in HUVECs. In addition, we also provided evidence that the signalling pathways of PI3K/AKT and MAPK/ERK1/2 take part in hucMSC-exosome-induced VEGF regulation.

**Conclusion:**

Our data suggest that hucMSC-exosomes exert a vasculoprotective role in the setting of vein graft disease, which may provide a new clue to protect against vein graft failure in the future.

## Background

Cardiovascular disease (CVD) remains one of the most frequent causes of death in human beings. Coronary artery stenosis and occlusions manifest as both acute and chronic ischaemia, and these events occupy the predominant position of morbidity and mortality in cardiovascular disease. Coronary artery bypass graft surgery (CABG) with autogenous vein is the most widely used and recommended therapies for severe ischaemic heart disease [1]. However, the mid-long-term effectiveness of CABG remains affected by vein graft failure. Intimal hyperplasia of the vein grafts is considered to be the principal pathophysiological process responsible for vein graft failure, but there are still no effective clinical treatments [2].

During the past decade, MSCs have been widely studied in clinical settings for tissue repair and regeneration due to their multi-lineage differentiation potential [3]. There is accumulating evidence suggesting that beneficial effects of stem cell transplantation might be largely, or at least partially, caused by a paracrine mechanism [4, 5]. Exosomes, small membrane vesicles secreted from many kinds of cells, are an important part of paracrine secretion. They are positive for CD9, CD63, and CD81. They performed an important function in acellular modes of communication, leading to the intercellular transfer of molecules such as mRNAs, miRNAs, and proteins to adjacent cells or tissues to regulate physiological and pathological processes [6–9]. Recent evidences have indicated that MSC-exosomes have a similar therapeutic effect in tissue regenerating and repairing as in recipient cells from which they are secreted [9–12]. Besides, non-cell-based MSC-exosome therapy also decreases numerous complications related to cellular transplantation, such as immune reaction, malignant proliferation, and vascular embolization [4].

In our previous study, we have found that MSC transplantation after vein grafting can prevent intimal hyperplasia by accelerating reendothelialization in a rat vein graft model [13, 14]. This effect was, in part, attributable to MSCs homed to the site of injury and differentiated into an endothelial phenotype to re-establish the endothelial layer. However, the exact mechanisms of action of the MSC transplantation still need better understanding. We hypothesized that MSC-exosomes may also play a crucial role in intimal hyperplasia and endothelial functional recovery. In the present study, we hypothesized that MSC-exosomes could be used as a novel alternative for using MSCs in the treatment of vein graft disease.

## Materials and methods

### Isolation and characterization of hucMSCs and HUVECs

All people provided informed consent for the use of the umbilical cord in this experimental study, which was approved by the Ethical Committee of the Qilu Hospital of Shandong University (KYLL-2017-106). The primary hucMSCs and HUVECs were isolated and cultured following an established method. The hucMSCs were cultured in α-MEM (Hyclone, USA) in humidified air with 5% CO2. hucMSCs were identified by flow cytometry analysis using the following fluorescein antibodies: CD29, CD34, CD44, CD45, and CD90. The differentiative capacity of hucMSCs was measured using a adipogenic differentiation medium and chondrogenic differentiation medium (ChemBio, China). The HUVECs were maintained in ECM (ScienCell, USA) supplemented with 5% foetal bovine serum. HUVECs were identified by their cobblestone appearance and by von Willebrand factor (vWF) and CD31 immunofluorescence staining. All cells were subcultured using trypsin and used for experiments up to passage 5.

### Extraction and identification of hucMSC-exosomes

hucMSC-exosomes were extracted from the conditioned media of MSC by high-speed centrifugation according to the protocol described by Lee et al. [15]. In brief, the cell supernatants were centrifuged at 1500*g* for 5 min at room temperature to discard cellular debris, followed by centrifugation at 100,000×*g* for 1 h at 4 °C. The exosome pellets were resuspended in phosphate-buffered saline (PBS) and maintained at − 80 °C for use in subsequent experiments. These particles were visualized with a transmission electron microscopy (JEOL-1200EX, Japan). The diameter distribution of MSCExo was obtained by Image-Pro Plus based on the previous study reported by Bian et al. [16]. The specific exosome markers, including CD9, CD63, and CD81, were identified by western blot analysis.

### Rat vein graft model and treatment

All experimental procedures were approved by the Animal Care and Use Committees at Qilu Hospital of Shandong University. The model was carried out using the anastomotic cuff technique as we have described [13, 14, 17, 18]. Briefly, adult Wistar rats were prepared and made intraperitoneal anaesthesia with chloral hydrate. The jugular vein was autologously inserted into the infrarenal abdominal aorta using a 20-GA intravenous cannula (BD, Sweden) and ligated to two cuffs with 5-0 silk. We confirmed immediate restoration of blood flow upon removal of the arterial occlusion clamps. To eliminate the harmful effect of thrombus formation in the vein grafts, vascular ultrasound examination was performed during the whole observation period.

To investigate the effect of hucMSC-exosomes, Wistar rats were averagely divided into 3 groups: the normal vein group, vein graft + PBS group (200 μl PBS was infused via the tail vein), and vein graft + exosome group (400 μg hucMSC-exosome protein suspended in 200 μl PBS was infused). To evaluate the effect of exosomes on haemodynamics, the vein graft diameter and peak-systolic velocity (PSV) were measured before tissue harvesting by a small animal ultrasound scanner as we have described [18]. The rats were killed at 2 weeks and 4 weeks after surgery for histomorphometric analysis based on the different experiments.

### HE staining and immunohistochemical staining

Tissue segments of vein grafts harvested at 4 weeks were fixed in 4% paraformaldehyde, embedded in paraffin, and sectioned at 5 μm thickness. The sections were stained with haematoxylin and eosin (HE) to observe the structure and measure the neointimal thickness by Image-Pro Plus software. Matrix metalloproteinase-2 (MMP2, 1:200, Proteintech, Wuhan, China) and matrix metalloproteinase-9 (MMP9, 1:200, Proteintech) and proliferating cell nuclear antigen (PCNA, 1:10000, Abcam, UK) immunohistochemical staining was carried out using SP-9100 Detection Kits to figure out neointimal formation in the vein grafts. The PCNA proliferation index was defined as the percentage of the PCNA-positive cells in the neointima of each section.

### Immunofluorescence study

To assess reendothelialization, vein grafts harvested at 2 weeks were fixed in 4% paraformaldehyde, embedded in OCT, and sectioned into 10-μm-thick sections. The sections were incubated with primary antibodies of CD31(1:200, Abcam) and stained with secondary Alexa-Fluor-conjugated antibody (1:200, Proteintech). To identify primary cells isolated from the human umbilical cord, cells were incubated with vWF-FITC (1:50, Abcam) and CD31 (1:100, Abcam) and counterstained with DAPI. All the results were visualized by using a fluorescence microscope.

### hucMSC-exosomes uptake by HUVECs

hucMSC-exosomes were labelled with DiI dye as previously described [19]. The labelled hucMSC-exosomes were centrifuged at 100,000*g* to remove excess dye by precipitation of exosomes. DiI-labelled hucMSC-exosomes (10 μg/ml) were then incubated with HUVECs after the determination of the protein content. After incubation, cells were fixed in paraformaldehyde and nuclei were stained with DAPI. Cellular uptake of hucMSC-exosomes by HUVECs was observed using an inverted fluorescence microscope.

### Cell proliferation assay

The effects of hucMSC-exosomes on the proliferation of HUVECs were evaluated using the EdU incorporation assay kit (RiboBio, China) according to the instructions given by the manufacturer. After EdU staining, EdU-stained cells were counted under a fluorescence microscope in a blinded fashion. The proliferation rate was defined as the number of EdU-stained cells divided by the number of Hoechst 33342-stained cells.

### Migration assay

The effects of hucMSC-exosomes on HUVEC migration were evaluated in scratch assays. HUVECs were scratched with a sterile 200-μl pipette tip and different concentrations of exosomes were added to the wells. The images were recorded at 0 and 24 h after the monolayers were scratched. Cell migration was further assessed using a Boyden chamber as we described previously [13]. HUVECs were seeded in serum-free ECM in Transwell inserts, wherein the bottom chambers contained complete ECM and different concentrations of exosomes. The migration of cells was allowed to proceed for 24 h at 37 °C with 5% CO2. Cells that migrated to the bottom of the insert were fixed in 4% paraformaldehyde for 30 min and stained with 0.1% crystal violet for 15 min, and cell counts were performed via a light microscope. Scratched areas were measured using the Image-Pro Plus 6.0 software.

### Western blot

Western blot analysis was performed as we have previously described [18]. Blots were incubated with the appropriate primary antibodies, including CD9 (1:1000, Proteintech), CD63 (1:1000, Proteintech), CD81 (1:1000, BOSTER, Wuhan, China), inducible nitric oxide synthase (iNOS,1: 1000, Abcam), endothelial nitric oxide synthase (eNOS, 1:1000, Abcam), VEGF-A (1: 1000, Abcam), CD31 (1:1000, Abcam), AKT (1:1000,Cell Signaling, Boston, USA), p-AKT(,1:1000, Cell Signaling), ERK1/2 (1:1000, Cell Signaling), p-ERK1/2 (1:1000, Cell Signaling), and GAPDH (1:2000, Proteintech). The secondary antibody was horseradish peroxidase-conjugated anti-rabbit immunoglobulin IgG (1:5000, Proteintech). Immunoreactive bands were visualized by ECL substrate and performed using Image J software.

### qRT-PCR

Total RNA was isolated using TRIzol Reagent (Thermo Fisher, MA, USA), levels of eNOS and iNOS mRNA were measured using a PCR Kit (Toyobo, Osaka, Japan) according to the manufacturer’s specifications, and GAPDH was selected as the housekeeping gene for normalization. Expression levels were calculated using the ∆∆Ct method as we have previously described [13, 18].

### Statistical analysis

SPSS 20.0 software (Chicago, IL, USA) was used to perform with statistical analysis. All of the data in the study were expressed as mean ± SD. Statistical comparisons were conducted with Student’s *t* test and one-way ANOVA followed by the SNK test. *P* < 0.05 were considered statistically significant. Each experiment was repeated at least three times.

## Results

### Characterization of hucMSCs and hucMSC-exosomes

**Fig. 1:**
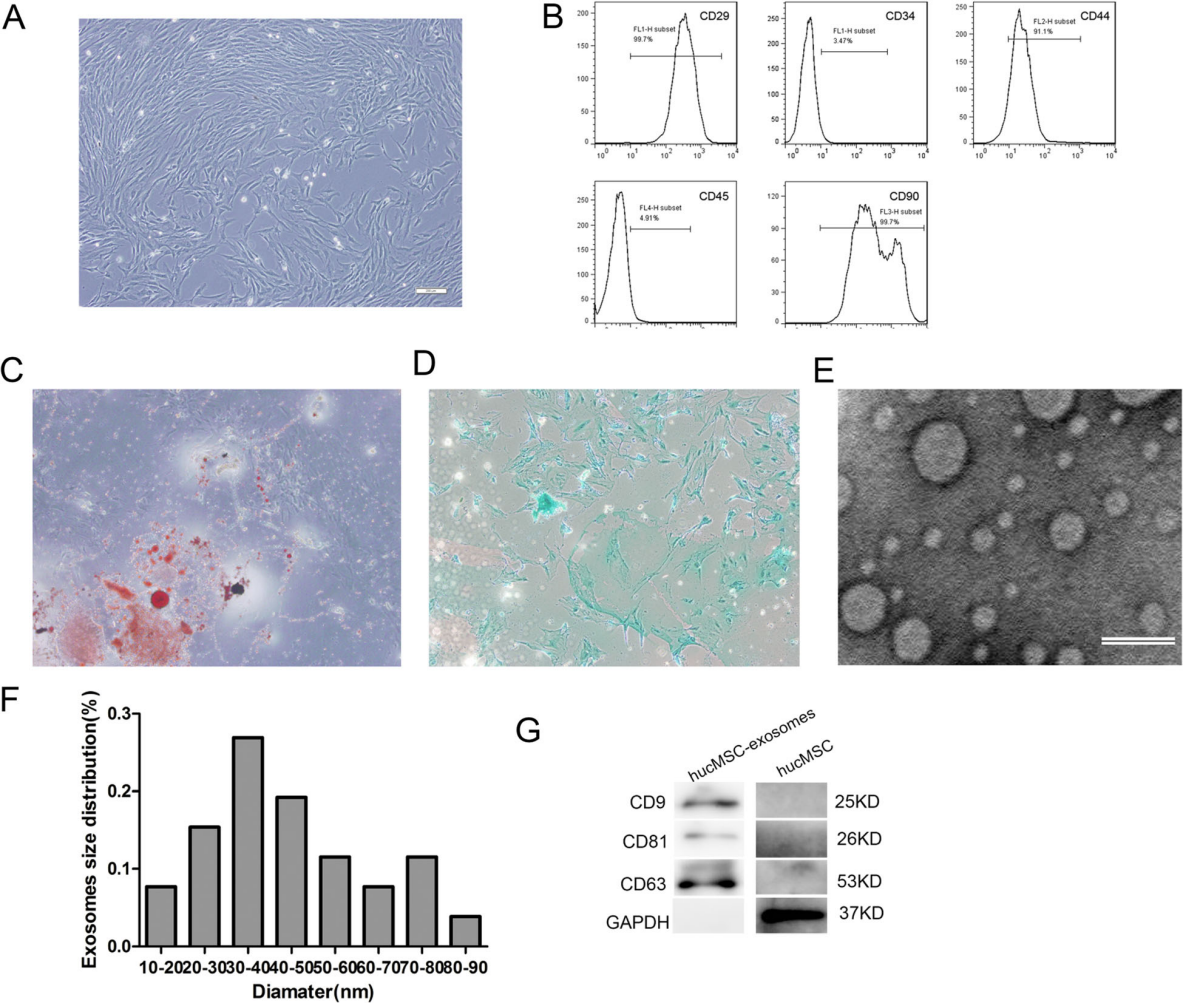
Morphology and characterization of hucMSCs and hucMSC-exosomes. **a** The fibroblast-like morphology of hucMSCs shown in light microscopy images (× 40). **b** Surface markers of hucMSCs analysed by flow cytometry. hucMSCs were positive for CD29, CD44, and CD90 and were negative for CD34 and CD45. **c** MSCs displayed the ability of adipogenic differentiation (× 40). **d** MSCs displayed the ability of chondrogenic differentiation (× 40). **e** Morphology of exosomes under transmission electron microscopy. Scale bar, 100 nm. **f** The size distribution of exosomes measured by Image-Pro Plus software. **g** Western blotting analyses of the exosome surface markers (CD9, CD81, and CD63)

The morphological characteristics of hucMSCs were seen under a light microscope, and all cells showed typical fibroblast morphology in vitro (Fig. 1a). Flow cytometry analyses showed that these cells were positive for CD29, CD44, and CD90 but were persistently negative for CD45 and CD34 (Fig. 1b), as previously reported. The potential of adipogenic differentiation and chondrogenic differentiation of hucMSCs were confirmed by Alcian blue staining and Oil Red O staining after 2–3 weeks of differentiation (Fig. 1c, d). The results proved that MSCs were successfully separated from the human umbilical cord. To verify the harvested exosomes, the morphology of exosomes was observed under a transmission electron microscope. They exhibited a round-shaped morphology, and they varied in size (Fig. 1e). The mean diameter was 44.54 ± 18.55 nm, which was the same with the previous research (Fig. 1f) [20]. Western blotting revealed the expression of CD9, CD63, and CD81 (Fig. 1g). In conclusion, the results indicated that these particles were actually exosomes.

### hucMSC-exosomes significantly inhibited neointimal hyperplasia and luminal stenosis in arterialized vein grafts

**Fig. 2:**
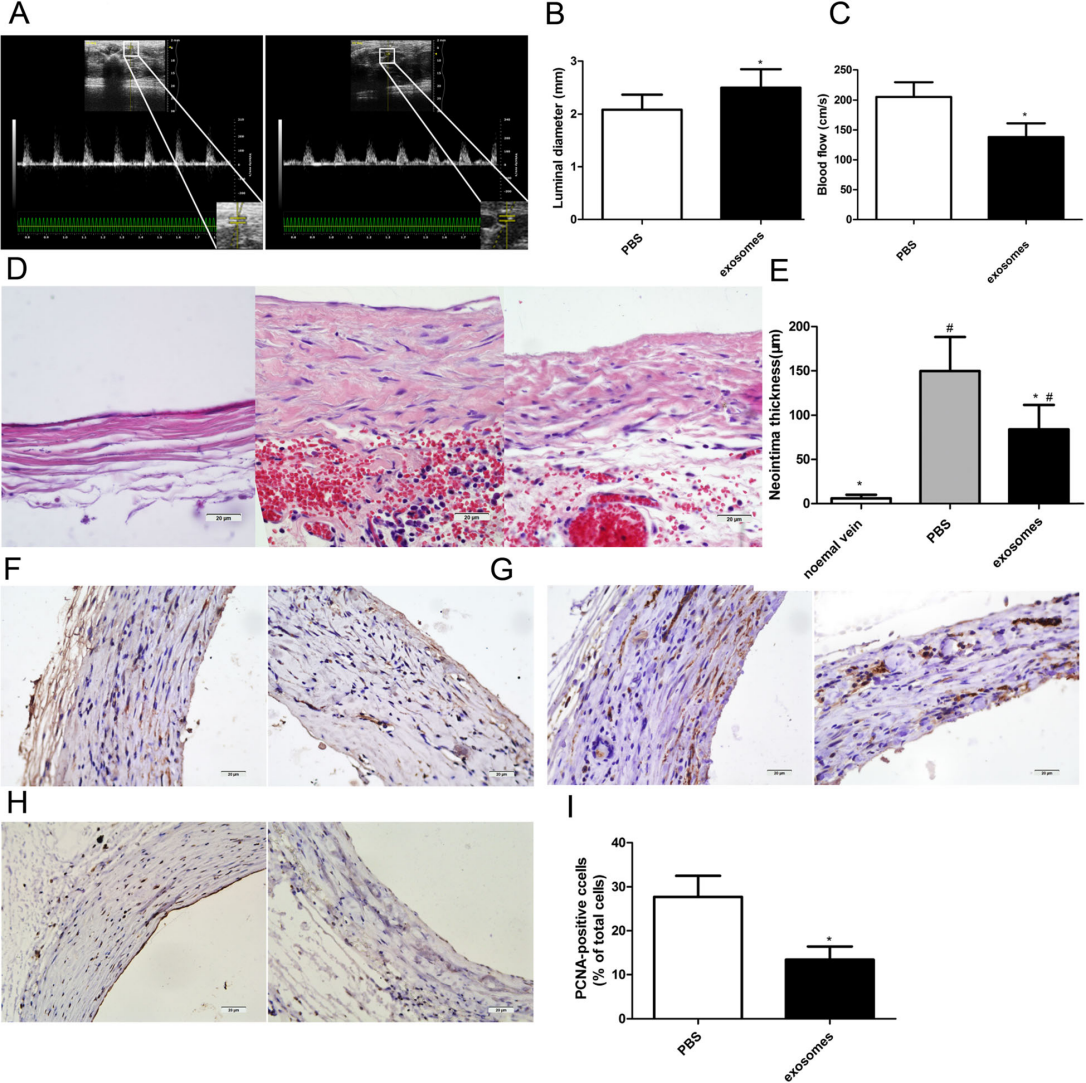
hucMSC-exosomes inhibited intimal hyperplasia and luminal stenosis in arterialized vein grafts. **a** Representative ultrasound images of vein grafts from the PBS group (left panel) and exosome group (right panel). Quantification of the luminal diameter (**b**) and peak-systolic velocity (**c**) in vein grafts. **d** Histologic images of haematoxylin and eosin staining (HE) staining in vein grafts from the normal vein group (left panel), PBS group (middle panel), and exosome group (right panel). **e** Quantification of the neointimal thickness in vein grafts. **f** Immunohistochemical staining of matrix metalloproteinase 2 (MMP-2) in vein grafts from the PBS group (left panel) and exosome group (right panel). **g** Immunohistochemical staining of matrix metalloproteinase 9 (MMP-9) in vein grafts from the PBS group (left panel) and exosome group (right panel). **h** Immunohistochemical staining of proliferating cell nuclear antigen (PCNA) in vein grafts from the PBS group (left panel) and exosome group (right panel). **i** Quantitative analysis of PCNA-positive index in vein grafts. The PCNA-positive index was quantified as the percentage of total nuclei in the neointima. Scale bar is 200 μm in panels **d**, **f**, **g**, and **h**. The results are presented as the mean ± SD, *n* = 6 for each group. An asterisk represents statistically significant difference compared with the PBS group (*P* < 0.05). Number sign represents statistically significant difference compared with the normal vein group (*P* < 0.05)

All rats that recovered from the surgery survived and no evidence of thrombosis formation was noted in vein grafts during the entire period of observation. To evaluate the effect of hucMSC-exosomes on neointimal lesion formation and luminal stenosis after surgery, the diameter and PSV in the distal anastomosis of vein grafts were analysed 28 days after vein grafting (Fig. 2a). The values for luminal diameter in the PBS and exosome groups were 2.08 ± 0.29 mm and 2.5 ± 0.35 mm (*P* < 0.05,Fig. 2b), respectively, and those for PSV in the PBS and exosome groups were 205.3 ± 24.57 cm/s and 138.3 ± 22.85 cm/s (Fig. 2c), respectively. The luminal diameters in the PBS group significantly decreased to 83.2%, whereas the peak velocity of the arteries increased by 1.48 times compared with that of the exosome group. Vein grafts were harvested 28 days after implantation, and histological assessment was performed by HE staining (Fig. 2d). Morphometric analysis revealed that the administration of hucMSC-exosomes resulted in a significant decrease in the neointimal thickness compared with PBS treatment (84 ± 27.69 μm vs 149.7 ± 38.58 μm, *P* < 0.05, Fig. 2e).

Because matrix metalloproteinases (MMPs) are important agents in promoting SMC migration and subsequent extracellular matrix deposition (ECM) in neointima [21], we evaluated the expression of MMP-2 and MMP-9 in grafted veins by immunohistochemistry. As shown in Fig. 2f and Fig. 2g, MMP-2 and MMP-9 were diffusely distributed within the neointimal region in both the PBS group and exosome group, and staining intensity appeared to be greater in the PBS group than in the exosome group. Moreover, cell proliferation in the neointima was detected by PCNA immunohistochemical staining (Fig. 2h). Moreover, neointimal hyperplasia was further evaluated by PCNA immunohistochemical staining (Fig. 2h). Quantitative analysis of the PCNA-positive cell index indicated the administration of hucMSC-exosomes decreased the number of PCNA-positive cells in the neointima compared with PBS treatment (13.43 ± 2.99% vs 27.71 ± 4.79%, *P* < 0.05, Fig. 2i). Together, these results showed that hucMSC-exosome administration significantly inhibited the neointimal lesion formation and luminal stenosis in arterialized vein grafts.

### hucMSC-exosomes significantly accelerated reendothelialization in arterialized vein grafts

**Fig. 3:**
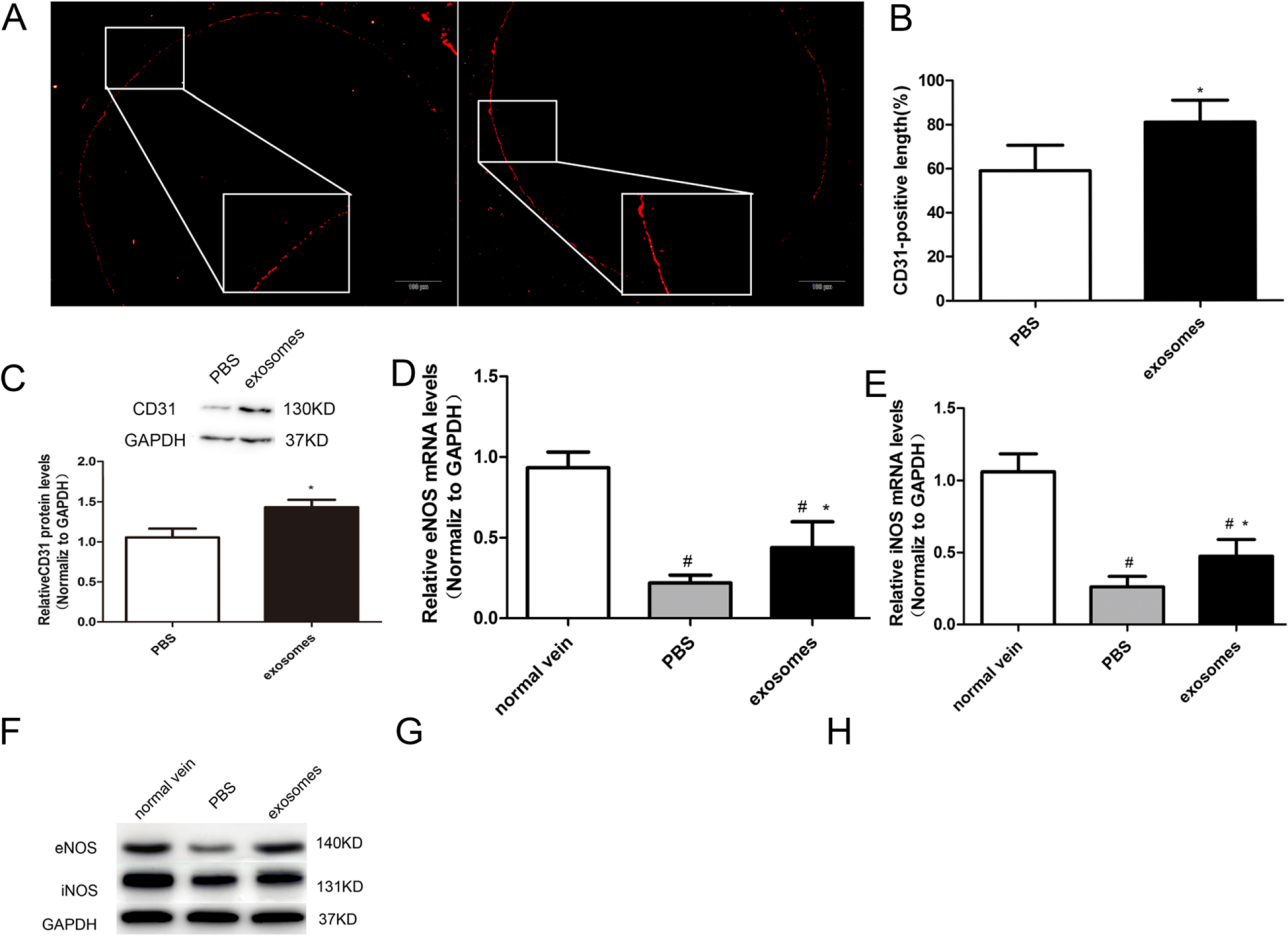
hucMSC-exosomes accelerated reendothelialization in arterialized vein grafts. **a** Immunohistochemical staining of vascular endothelial cell marker CD31 (red) in vein grafts from the PBS group (left panel) and exosome group (right panel) to assess endothelial recovery. Scale bar is 200 μm. **b** Quantitative analysis of reendothelialization in vein grafts at 14 days after vein grafting. Reendothelialization was expressed as a percentage of the CD31-positive stained luminal surface to the total luminal surface. **c** Representative western blots (top panel) and quantitative analysis (lower panel) of CD31protein expression in vein grafts from the PBS and exosome groups. The mRNA levels of eNOS (**d**) and iNOS (**e**) in vein grafts from the normal vein, PBS, and exosome groups were detected by RT-PCR. **f** Representative western blots of eNOS and iNOS protein expression in vein grafts from the normal vein, PBS, and exosome groups. Effects of hucMSC-exosome administration on eNOS (**g**) and iNOS (**h**) protein expression were quantified. The results are presented as the mean ± SD, *n* = 6 for each group. An asterisk represents statistically significant difference compared with the PBS group (*P* < 0.05). Number sign represents statistically significant difference compared with the normal vein group (*P* < 0.05)

To investigate if hucMSC-exosomes promote endothelial growth, immunofluorescence analysis of an endothelial cell marker (CD31) was performed at 2 weeks after vein grafting. As shown in Fig. 3a, CD31-positive immunostaining was observed along the luminal surface of the vein grafts. Quantitative analysis of the CD31-positive cells covering the luminal surface across the total length of the luminal surface revealed that reendothelialization in the exosome group was particularly greater than that in the PBS group (81 ± 10.02% vs. 59 ± 11.64%, *P* < 0.05, Fig. 3b). The western blot analysis of CD31 protein expression provided further evidence that reendothelialization in the exosome group was particularly greater than in the PBS group (Fig. 3c).

Nitric oxide (NO) has anti-inflammatory effects and protects against neointimal hyperplasia [22]. Therefore, we assessed the expression of NO synthase mRNA by RT-PCR. Quantitative analysis showed that both the relative mRNA expressions of eNOS and iNOS were decreased in the PBS group and exosome group compared to those of the normal vein group. Even the expression of eNOS and iNOS in vein graft was raised after treatment with exosomes, there were still significant differences between the exosome group and normal vein group (Fig. 3d, e). Furthermore, we detected the expression of eNOS and iNOS proteins using western blot (Fig. 3f). eNOS protein was obviously raised in the vein grafts of exosome-treated rats compared to that in PBS-treated rats (Fig. 3g). However, no significant difference in iNOS protein level was observed between the PBS group and exosome group (*P* = 0.28, Fig. 3h). All these results indicated that hucMSC-exosomes significantly accelerated reendothelialization in arterialized vein grafts.

### Characterization of HUVECs and internalization of hucMSC-exosomes by HUVECs

**Fig. 4:**
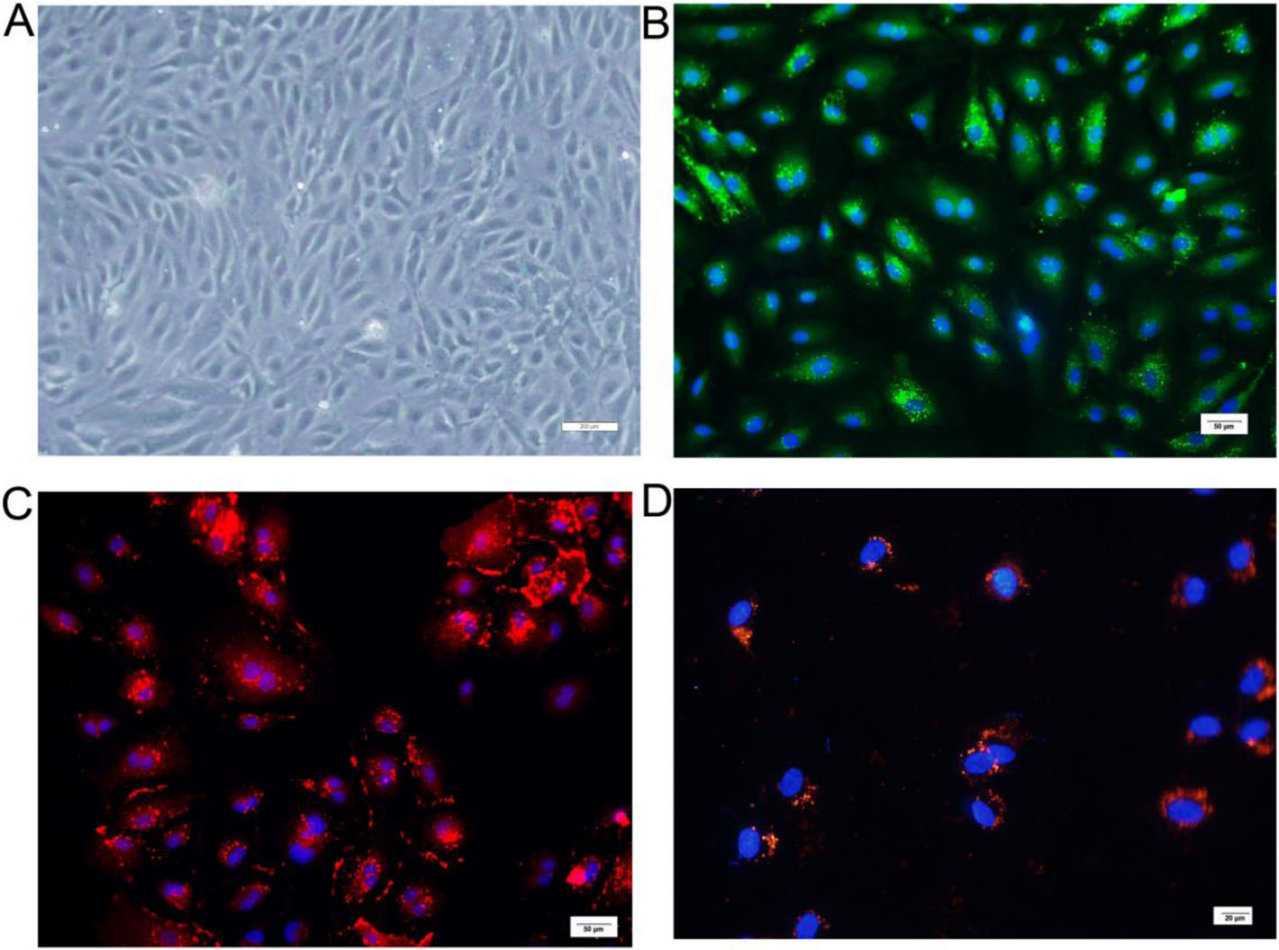
Morphology and characterization of HUVECs and cellular uptake of hucMSC-exosomes by HUVECs. **a** HUVECs cultured for 3 passages exhibited the typical cobblestone morphology of ECs. Bar, 200 μm. **b** HUVECs were identified by immunofluorescence staining for vWF (green). Nuclei were stained blue with DAPI. Bar, 50 μm. **c** HUVECs were identified by immunofluorescence staining for CD31 (red). Nuclei were stained blue with DAPI. Bar, 50 μm. **d** Cellular internalization of hucMSC-exosomes into HUVECs. DiI-labelled hucMSC-exosomes (red) were internalized into DAPI-labelled HUVECs (blue). Bar, 20 μm

To further verify the role of hucMSC-exosomes on endothelial function, we isolated HUVECs from fresh human umbilical cords. HUVECs cultured for 3 passages exhibited the typical cobblestone morphology of endothelial cells (Fig. 4a). Immunostaining confirmed that all the adherent cells were positive for CD31 and vWF (Fig. 4b, c). The results suggested that HUVECs had been successfully separated from the human umbilical cords. We then texted whether hucMSC-exosomes can be internalized into HUVECs. The red fluorescence in exosomes was localized in the cytoplasm of HUVECs, indicating that DiI-labelled hucMSC-exosomes can be internalized by HUVECs (Fig. 4c).

### hucMSC-exosomes promoted proliferation and migration of HUVECs in vitro

**Fig. 5:**
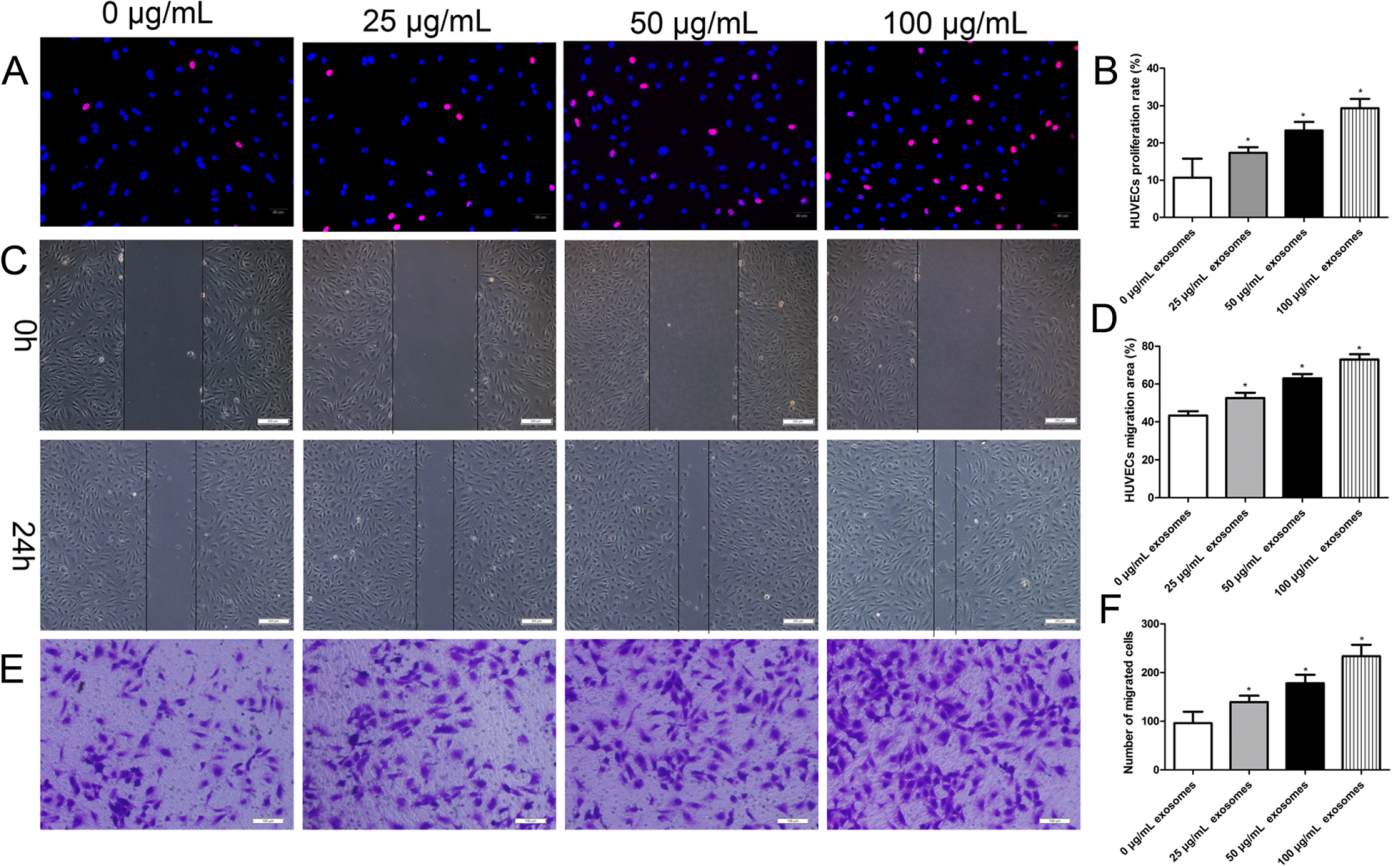
hucMSC-exosomes promoted in vitro proliferation and migration of HUVECs in a dose-dependent manner. HUVECs were incubated in the presence of different concentrations of hucMSC-exosomes (25, 50, and 100 μg/ml) for 24 h. **a** Representative images of the EdU incorporation assay of HUVECs. EdU-positive cells were red-stained, and negative cells had blue nuclei (Hoechst 33342). Bar, 50 μm. **b** Quantification of proliferation rates (percentage of EdU-positive cells) of HUVECs showed that the proliferative capacity of HUVECs was significantly improved dose-dependently. **c** Representative images of the scratched wound assay. Bar, 200 μm. **d** Quantitative analysis of the migration area of HUVECs showed that the migratory capacity of HUVECs was significantly improved dose-dependently. **e** Representative images of the Transwell assay of HUVECs. Bar, 100 μm. **f** Quantification of the number of migrated cells HUVECs showed that the migratory capacity of HUVECs was significantly improved dose-dependently. The results are presented as the mean ± SD, *n* = 3 for each group. An asterisk represents statistically significant difference compared with the control group (*P* < 0.05)

To study the role of hucMSC-exosomes on the proliferative and migratory activity of endothelial cells, HUVECs were treated with a series of concentrations of hucMSC-exosomes (0, 25, 50, and 100 μg/ml), and in vitro migration and proliferation experiments were performed. The function of hucMSC-exosomes on the proliferation of endothelial cells was assessed by EdU incorporation assay. The experimental data revealed that hucMSC-exosomes enhanced the proliferative capacity of HUVECs in a dose-dependent manner (Fig. 5a). The scratch wound assay and Transwell assay were used to investigate the function of hucMSC-exosomes on the migratory capacity of endothelial cells. Using the scratch wound assay, we found that HUVECs exhibited an enhanced migratory capacity after treatment with hucMSC-exosomes (Fig. 5b). As expected, the Transwell migration assay found that hucMSC-exosomes could enhance HUVEC migratory activity (Fig. 4c). All in all, the results demonstrated that hucMSC-exosomes promoted HUVEC proliferative and migratory activities, suggesting important roles in vascular reendothelialization. These results were consistent with the in vivo data.

### VEGF played a critical role in hucMSC-exosome-mediated promotion of proliferation and migration in HUVECs

**Fig. 6:**
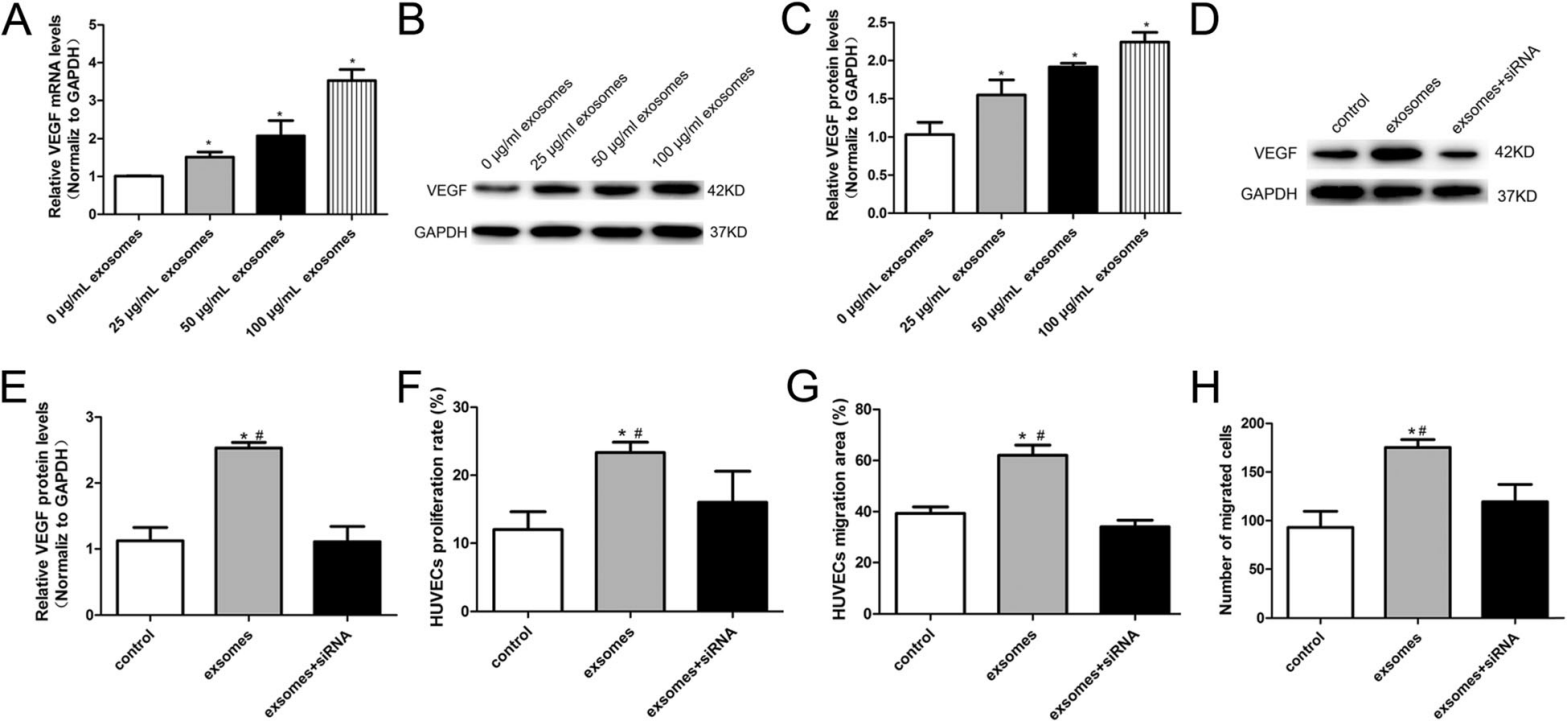
VEGF played a critical role in hucMSC-exosome-mediated promotion of proliferation and migration in HUVECs. **a** hucMSC-exosomes increased VEGF mRNA expression measured by RT-PCR in a dose-dependent manner in HUVECs. **b** Representative western blot and analyses (**c**) of VEGF protein expression showed hucMSC-exosomes increased VEGF protein level in a dose-dependent manner in HUVECs. **d** Representative western blot and analyses (**e**) of VEGF protein expression showed hucMSC-exosomes could not increase the protein levels of VEGF after co-culture with VEGF siRNA.**f** HUVECs were transfected with VEGF siRNA and incubated with hucMSC-exosomes, and quantitative analysis of EdU incorporation assay showed that the enhanced proliferative ability induced by hucMSC-exosomes was abolished by VEGF siRNA. **g** HUVECs were transfected with VEGF siRNA and incubated with hucMSC-exosomes. The quantitative analysis of scratched wound assay showed that the enhanced migratory ability induced by hucMSC-exosomes was abolished by VEGF siRNA. **h** HUVECs were transfected with VEGF siRNA and incubated with hucMSC-exosomes, and quantitative analysis of Transwell assays showed that the enhanced migratory ability induced by hucMSC-exosomes was abolished by VEGF siRNA. VEGF siRNA, 100 nM; hucMSC-exosomes, 50 μg/ml. The results are presented as the mean ± SD, *n* = 3 for each group. An asterisk represents statistically significant difference compared with the control group (*P* < 0.05). Number sign represents statistically significant difference compared with the hucMSC-exosomes + VEGF siRNA group (*P* < 0.05)

VEGF is well-known to modulate endothelial cell proliferation and migration both in vivo and in vitro. Therefore, we guessed whether there was some relationship between hucMSC-exosomes and VEGF expression in our research. Our group first evaluated the expression of VEGF in HUVECs treated with hucMSC-exosomes by RT-PCR. We found that hucMSC-exosomes increased VEGF mRNA levels in a dose-dependent manner (Fig. 6a). In addition, the VEGF protein expression was detected by western blot. Consistently, the incubation of HUVECs with hucMSC-exosomes resulted in increased expression of VEGF in a dose-dependent manner (Fig. 6b, c).

To further investigate that the effect of hucMSC-exosomes on proliferation and migration of HUVECs is mediated by the regulation of VEGF, HUVECs were transfected with VEGF siRNA (100 nM) and delivered with hucMSC-exosomes (50 μg/ml). The results of the western blot suggested that the VEGF expression was significantly lower in hucMSC-exosomes + VEGF siRNA group than in the hucMSC-exosome group (Fig. 6d, e). We next checked whether hucMSC-exosomes could still stimulate proliferative and migratory activities following the application of VEGF siRNA on HUVECs. The result of proliferation and migration assays showed that the proliferative and migratory activities were reduced in endothelial cells treated with hucMSC-exosomes + VEGF siRNA compared with the cells transfected with hucMSC-exosomes (Fig. 6f–h). All these dates indicated that VEGF plays an important role in hucMSC-exosome-mediated promotion of proliferative and migratory activities in HUVECs.

### PI3K/AKT and MAPK/ERK1/2 signalling pathways are involved in hucMSC-exosome-induced VEGF upregulation

**Fig. 7:**
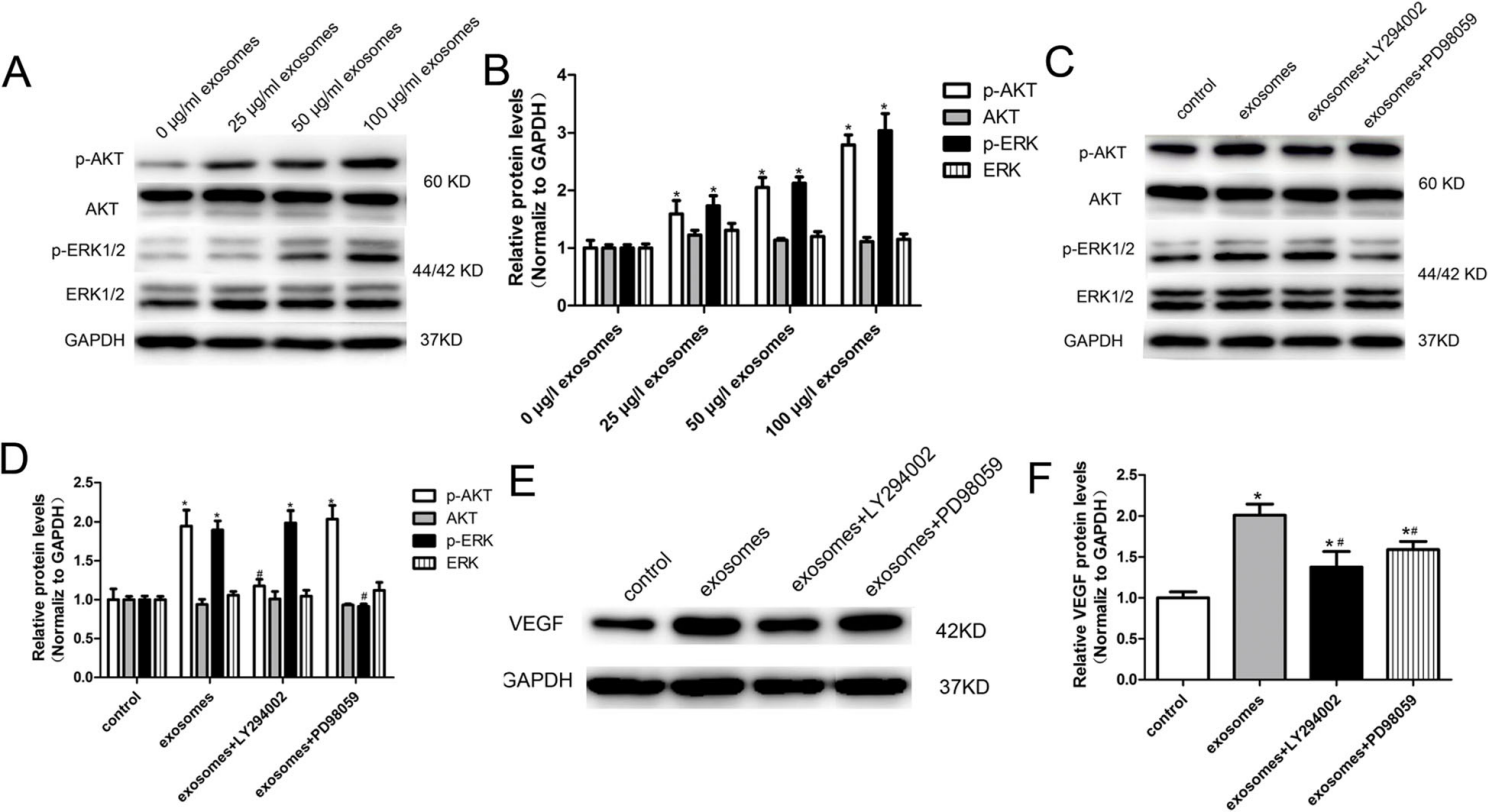
PI3K/AKT and MAPK/ERK1/2 signalling are involved in hucMSC-exosome-induced VEGF regulation. **a** Representative image of western blot analysis of ERK and AKT phosphorylation level in HUVECs treated with various concentrations of exosomes (0, 25, 50, and 100 μg/ml). **b** hucMSC-exosomes increased phosphorylation of AKT and ERK1/2 as measured by western blotting analysis. **c** Representative image of western blot analysis of ERK and AKT phosphorylation levels in HUVECs treated with hucMSC-exosomes and AKT inhibitor LY294002 and ERK1/2 inhibitor PD98059. **d** AKT inhibitor LY294002 and ERK1/2 inhibitor PD98059 decreased hucMSC-exosome-induced phosphorylation of AKT and ERK in HUVECs. **e** Representative image of western blot analysis of VEGF protein expression in HUVECs treated with hucMSC-exosomes and AKT inhibitor LY294002 and ERK1/2 inhibitor PD98059. **f** AKT inhibitor LY294002 and ERK1/2 inhibitor PD98059 significantly inhibited hucMSC-exosome-induced VEGF protein expression. VEGF siRNA, 100 nM; hucMSC-exosomes, 50 μg/ml; LY294002, 20 μM; PD098059, 20 μM. The results are presented as the mean ± SD, *n* = 3 for each group. An asterisk represents statistically significant difference compared with the control group (*P* < 0.05). Number sign represents statistically significant difference compared with the hucMSC-exosome group (*P* < 0.05)

Next, we tried to figure out the signalling pathways that are involved in hucMSC-exosome-induced VEGF upregulation. Because the PI3K/AKT and MAPK/ERK1/2 signalling pathways are essential for cell biological activities and are associated with VEGF regulation [23–25], we assessed AKT and ERK1/2 activity in HUVECs by western blot (Fig. 7a). Experimental results show that hucMSC-exosome induction did not change AKT and ERK1/2 expression levels, but strengthened the phosphorylation levels of AKT and ERK1/2 in HUVECs (Fig. 7b). Moreover, AKT phosphorylation and ERK1/2 phosphorylation levels were increased in a dose-dependent manner with increasing hucMSC-exosome concentrations (Fig. 7b). These data suggested that hucMSC-exosomes could activate both PI3K/AKT and MAPK/ERK1/2 signalling pathways to exert biological effects in HUVECs.

To further confirm that the activated PI3K/AKT and MAPK/ERK1/2 signalling pathways were associated with hucMSC-exosome-induced VEGF expression, HUVECs were pretreated with LY294002 (AKT inhibitor, 20 μM) or PD098059 (ERK1/2 inhibitor, 20 μM) prior to hucMSC-exosome treatment, and protein levels of AKT p-AKT, ERK, and p-ERK were assessed by western blot (Fig. 7c). As in the previous results, hucMSC-exosomes increased phosphorylation of AKT and ERK1/2 levels in HUVECs (Fig. 7d). However, AKT and ERK1/2 inhibitors almost completely blocked this effect. There was no obvious effect of LY294002 or PD098059 on AKT and ERK1/2 protein levels (Fig. 7d). This suggested that hucMSC-exosomes could not activate the PI3K/ AKT and MAPK/ERK1/2 signalling pathway after the pathway was blocked. We next evaluated whether hucMSC-exosomes could still increase VEGF expression following the application of LY294002 and PD098059 in HUVECs. The results showed that both AKT inhibitor LY294002 and ERK1/2 inhibitor PD098059 remarkably reduced hucMSC-exosome-induced VEGF upregulation (Fig. 7e, f). These findings confirmed that hucMSC-exosomes induce VEGF expression by acting downstream of the PI3K/AKT and MAPK/ERK1/2 signalling pathways.

## Discussion

Regardless of the many advances in the treatment of cardiovascular disease, there are no effective treatment or prevention methods to control vein graft failure. Poor long-term patency of saphenous veins remains an obstacle in CABG using an autologous vein graft to treat severe coronary artery stenosis. The present study suggests, for the first time, that hucMSC-exosomes could inhibit vein graft intimal hyperplasia and accelerate reendothelialization in a rat vein graft model. It also provides the first evidence that the hucMSC-exosomes stimulate proliferation and migration of vascular endothelial cells through activation of PI3K/AKT and MAPK/ERK1/2 /VEGF signalling pathways in vitro.

MSCs have been described to be a perfected choice for tissue repair and regeneration in coronary heart disease, heart failure, and pulmonary hypertension [26–28]. Currently, more and more evidence has indicated that the benefit of stem cell treatment might be through extracellular vesicle-mediated mechanisms [4, 5]. Stem cell-derived exosomes have been considered central mediators of cell-cell communication even across the species barriers [24]. In fact, stem cell-derived exosome-mediated cross-species communication is being directly used as therapeutic agents and has been proved in various animal models of CVD.

Recent studies from two different groups reported that exosomes derived from both human cardiac progenitor cells and embryonic stem cells can enhance cardiac myocyte repair and improve heart function in a MI model [29, 30]. Lee et al. proved that the treatment of hucMSC-exosomes limits lung inflammation, vascular remodelling and right heart failure in a murine model of hypoxic pulmonary hypertension [15]. Another study showed exosomes from human CD34+ stem cells increasing proliferative and migratory and tube forming activity of endothelial cell in vitro and stimulated angiogenesis in vivo [31]. Similar results were proved by Mathiyalaga et al. in mice ischaemic hindlimb model [32]. Chen et al. discovered that exosomes secreted by human endothelial progenitor cells could enhance endothelial function by regulating angiogenesis-related genes in ECs, promoting vascular repair, and accelerating reendothelialization in a rat model of balloon injury in the artery wall [33]. However, there is limited knowledge of the role of exosomes associated with vein graft disease. As shown in our study, MSC transplantation could efficiently inhibit vein graft intimal hyperplasia by enhancing endothelial function in vascular injury [13, 14]. It has been proved that exosomes derived from MSCs have played similar functional roles to that of MSCs [34]. We then further investigated whether systematic administration of hucMSC-exosomes could be of potential therapeutic benefit in vein graft disease and to identify their related molecular mechanisms in this study.

It is known that the mechanism causing neointimal hyperplasia involves a lot of cellular and molecular processes [35]. Ischaemia of the vein grafts and injured and denudated vein graft endothelium can induce platelet adhesion and inflammatory cell infiltration in the endothelial surface as well as vasospasm resulting from decreased NO levels [36]. Endothelial dysfunction, subsequent uncontrolled SMC proliferation, extensive ECM deposition, inflammatory cells, and inflammatory cytokines all exacerbate neointimal hyperplasia [37, 38]. To investigate whether intravenous administration of exosomes derived from hucMSCs has the same efficacy of MSCs on neointimal hyperplasia of vein grafting in a rat vein graft model, we performed a vascular ultrasound examination to evaluate blood flow and vascular stenosis and HE staining to measure the neointimal thickness. Moreover, immunohistochemistry for MMP2 and MMP9 and PCNA were also processed to assess neointimal hyperplasia level. All these experiments proved that hucMSC-exosome exhibited a beneficial effect on vascular repair in the rat vein graft model. The intact endothelial monolayer is important for the vessel wall to keep the integrity and function, to provide a barrier to limit the proliferation and migration of SMCs and ECM deposition, and to promote the synthesis of vasoprotective mediators such as NO [38].Therefore, we hypothesized that the beneficial effect of exosomes on neointimal hyperplasia may be partly owed to the rapid reendothelialization and recovery of endothelial function. Subsequent experiments of CD31 immunofluorescence staining and quantification of eNOS and iNOS expression in the vessel wall fully confirmed our hypothesis. It was reported that adenovirus-mediated gene transfer of NOS in porcine vein grafts reduces intimal thickening [39]. It is possible that the success of hucMSC-exosome treatment in inhibiting vein graft intimal hyperplasia may be partly owing to the vascular endothelial cell secretion of substances such as NO, which have vasoprotective properties [37, 40]. In our study, we found a statistical difference of eNOS, but not iNOS, protein expression between the PBS group and exosome group, which need to be further investigated.

Enhanced reendothelialization is beneficial in preventing vein graft failure, and the damaged vein graft depends on the proliferative and migratory activity of endothelial cells to promote reendothelialization and endothelial functional recovery [2]. As seen in vitro, membrane dye labelling proved that hucMSC-exosomes can be assimilated into HUVECs. This is similar to the report showing the incorporation of exosomes secreted by adipose-derived MSCs into HUVECs [41]. As it is known that VEGF is specifically considered as an important EC activator and regulates a variety of EC physiological functions including proliferation and migration [42], VEGF gene transfer could attenuate intimal hyperplasia by upregulating NO expression [43, 44]. We next examined whether there are any relationships between VEGF expression and hucMSC-exosome-induced endothelial cell proliferative and migratory activity. Interestingly, the results showed that hucMSC-exosomes upregulated the mRNA and protein levels of VEGF in HUVECs. Furthermore, we also proved that the increased amount of VEGF by hucMSC-exosomes was directly connected to the promotion of endothelial cell proliferation and migration. The results were somewhat analogous to a recent report that exosomes derived from hbmMSCs raise VEGF expression in SGC-7901 and SW480 tumour cells, resulting in enhanced capacity for proliferation and metastasis of tumours in vivo [45].

The PI3K/AKT and MAPK/ERK1/2 are regulators of cell biological activities and were described to be associated with VEGF regulation [23–25]. Guo et al. reported that PI3K/AKT and ERK1/2 are involved in exosomes derived from platelet-induced angiogenesis, which promote proliferation and migration effects in fibroblasts [24]. Zhu et al. demonstrated that PI3K/AKT and MAPK/ERK1/2 played a part in insulin-like growth factor-1-induced VEGF upregulation in breast cancer [23]. Thus, we evaluated protein levels of AKT, p-AKT, ERK, and p-ERK by western blot. Our data showed that AKT phosphorylation and ERK1/2 phosphorylation were increased with increasing hucMSC-exosome concentrations. Importantly, hucMSC-exosome-enhanced ERK and AKT activation could be specifically blocked by ERK and AKT inhibitors. We further examined whether hucMSC-exosome-induced VEGF upregulation was relevant to increased phosphorylation levels of AKT and ERK1/2 induced by hucMSC-exosomes. Our data indicated that ERK and AKT inhibitors remarkably reduced hucMSC-exosome-induced VEGF upregulation. All the experiments demonstrated that hucMSC-exosomes stimulate VEGF-induced proliferation and migration of HUVECs via the PI3K/AKT and MAPK/ERK1/2 signal.

There are still a few unsolved questions related to the role of hucMSC-exosomes in neointimal hyperplasia and the mechanisms of endothelial functional recovery in our settings. Future experiments will be performed to figure out the key agents (miRNA, lncRNA, and proteins) contained in the exosome cargo accounting for the vasculoprotective effects of hucMSC-exosomes against vein graft failure. Future experiments will be examined if there are inflammatory cells participated in vein graft reendothelialization. Furthermore, later experiments will be examined if there are any other molecules except VEGF that induced endothelial functional recovery. In addition, Deeper researches are also needed to discuss the long-term effects of hucMSC-exosome transplantation in our experimental vein graft model and whether these small membrane vesicles could be used as a valid and safe therapeutic strategy for clinical applications.

## Conclusions

Taken together, our results have clearly demonstrated that hucMSC-exosomes inhibited vein graft neointimal hyperplasia and accelerated reendothelialization by enhancing endothelial function and that AKT and ERK1/2/VEGF signalling pathways played a part in hucMSC-exosome-induced endothelial functional enhancement. This study may provide a novel therapeutic strategy by which exosomes could be used as a promising non-cell-based alternative for using MSCs in the treatment of vein graft disease in the future.

## Data Availability

Not applicable.

## Abbreviations

MSC: Mesenchymal stem cell
hucMSCs: Human umbilical cord MSCs
hucMSC-exosomes: Exosomes derived from human umbilical cord MSCs
VEGF: Vascular endothelial growth factor
CVD: Cardiovascular disease
HUVEC: Human umbilical vein endothelial cell
FBS: Foetal bovine serum
vWF: von Willebrand factor
PBS: Phosphate-buffered saline
PSV: Peak-systolic velocity
HE: Haematoxylin and eosin
MMP2: Matrix metalloproteinase-2
MMP9: Matrix metalloproteinase-9
PCNA: Proliferating cell nuclear antigen
eNOS: Endothelial nitric oxide synthase
iNOS: Inducible nitric oxide synthase
MMPs: Matrix metalloproteinases
NO: Nitric oxide
NOS: Nitric oxide synthase
SMC: Smooth muscle cell
